# Synthesis of an *ent*-Halimanolide from *ent*-Halimic Acid

**DOI:** 10.3390/molecules13051120

**Published:** 2008-05-09

**Authors:** Isidro S. Marcos, Almudena Conde, Rosalina F. Moro, Pilar Basabe, David Díez, Faustino Mollinedo, Julio G. Urones

**Affiliations:** 1 Departamento de Química Orgánica, Universidad de Salamanca, Plaza de los Caídos 1-5, 37008 Salamanca, Spain; 2 Centro de Investigación del Cáncer, Instituto de Biología Molecular y Celular del Cáncer, Consejo Superior de Investigaciones Científicas-Universidad de Salamanca, Campus Miguel de Unamuno, E-37007 Salamanca, Spain.; 3 APOINTECH, Campus Miguel de Unamuno, E-37007 Salamanca, Spain

**Keywords:** *Ent*-halimanolides, *ent*-halimic acid, diterpenoids, *Cladogynos orientalis*

## Abstract

An efficient synthesis of *ent*-halimanolide **2** (15,16-epoxy-12-oxo-*ent*-halima-5(10),13(16),14-trien-18,2β-olide), from *ent*-halimic acid has been achieved, corroborating the structure of the natural compound and establishing its absolute configuration.

## Introduction

Euphorbiaceae plants are a rich source of bioactive substances [1,2] and certain genera of this family have attracted much interest, since they contain a group of antitumor compounds [3]. *Cladogynos orientalis* Zipp. ex Span. (syn. *Adenochlaena siamensis* Ridl.) (Euphorbiaceae), known in Thailand as “Chettaphangki,” is the only member of the genus *Cladogynos* and the roots are used as a carminative in Thai folk medicine. Chettaphanin I [4,5] and II [6,7], are the main components from their roots of this plant and the first to be known. Recently, in addition to chettaphanin I and II, isolation from the root extract of a series of furan diterpenes **2**-**4 **with *ent*-halimane skeletons has been described [8].

**Figure 1:**
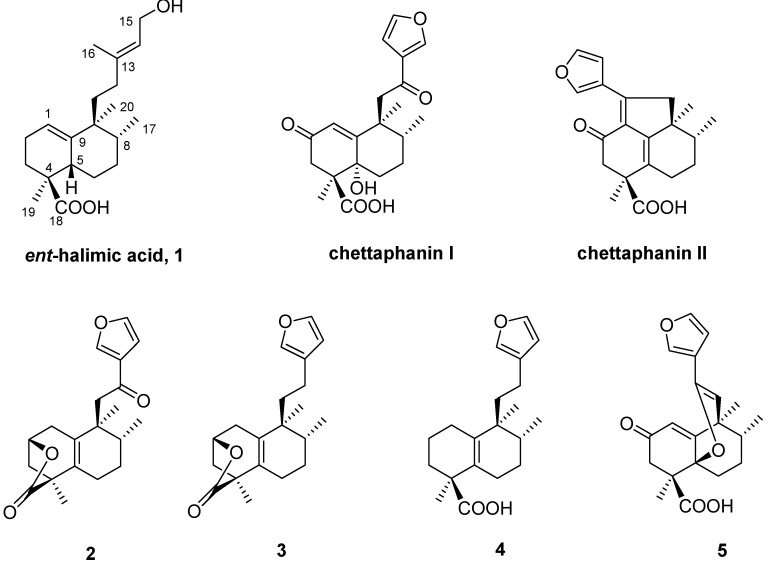
Structures of furo-*ent*-halimanes from *Cladogynos orientalis* and *ent*-halimic acid.

Our group has transformed *ent*-halimic acid **1**, a diterpene of known structure and absolute configuration, into chettaphanin I and II, which confirmed their structure and absolute configuration [4,6]. In this paper, we report the synthesis of *ent*-halimanolide **2**, in order to confirm the structure of the natural compound and do SAR studies. *ent*-Halimanolide is a furan diterpene like chettaphanin I and II, but in this case the carboxylic acid at C-18 has formed a γ-butanolide with the hydroxyl at C-2.

## Results and Discussion

In order to synthesise compound **2**, from *ent*-halimic acid **1** it is necessary to functionalize C-2 and C-12, add a furan group in the side chain, isomerize the double bond to the more stable tetrasubstituted position and to form the lactone ring.

Two synthetic routes have been explored for the synthesis of **2**: Route A and Route B, which differ in the strategy followed for the preparation of the γ-lactone, before or after of the introduction of the furan ring.

**Scheme 1:**
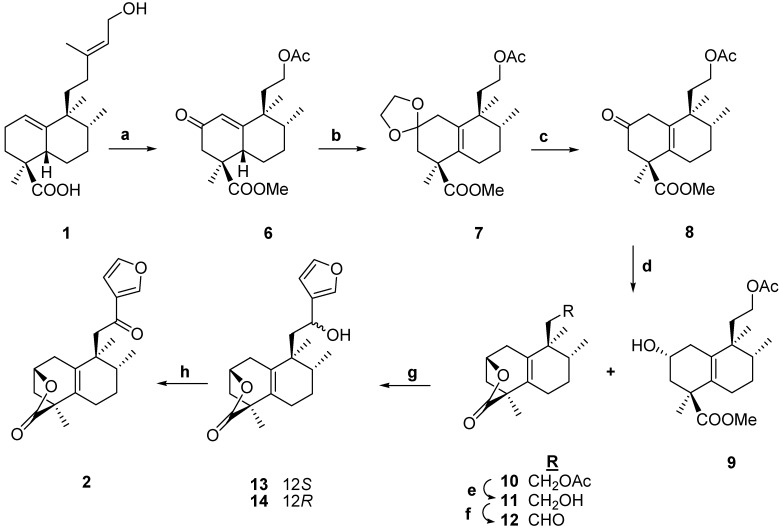
a) Ref. 3a; b) ethylene glycol, *p*-TsOH, C_6_H_6_, reflux, 8h (97%); c) HCl 2M, EtOH, 3h (92%); d) NaBH_4_, EtOH, rt, 3h (**9**: 47%, **10**: 49%); e) Na_2_CO_3_, MeOH, rt, 5 h (97%); f) TPAP, NMO, DCM, rt, 30 min (96%); g) 3-bromofuran, *n*-BuLi (1/1), -78ºC, 30 min (**13** : 54%, **14** : 36%); h) TPAP, NMO, DCM, rt, 50 min (92%).

In Route A (Scheme 1) three fundamental parts can be differentiated: elaboration of the adequate *ent*-halimic acid tetranorderivative **9**; γ-lactone formation as **12**, necessary for the final introduction of the furan fragment and to prepare the functional groups required to achieve the natural product **2**.

The *ent*-halimic acid tetranorderivative **6** was obtained in excellent yield as described in the synthesis of chettaphanin I and II [4] [6]. Carbonyl protection of **6** with ethylene glycol in acid media gives the tetranorderivative **7**, that already contains the tetrasubstituted double bond in the required position. The carbonyl deprotection should be done very carefully (controlling the acid and time) and in this manner ketone **8 **can be obtained, which by NaBH_4 _reduction gives a 1:1 mixture of hydroxyderivative **9** and γ-lactone **10**.

The required aldehyde **12** was obtained by hydrolysis of **10** followed by TPAP [9] oxidation of the hydroxy derivative **11**. Addition of 3-furyl lithium [10,11,12] to **12** gives a mixture of the hydroxy derivatives **13** and **14**. The C-12 configuration in **13** and **14** was established by comparation of their physical properties with the ones of similar compounds [13,14]. Oxidation of the mixture of **13** and **14** with TPAP gives **2**, 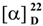 -101.4 (c 0.2, CHCl_3_), that was identical in all its physical properties to the natural compound 15,16-epoxy-12-oxo-*ent*-halima-5(10),13(16),14-trien-18,2β-olide, 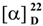 -151.5 (c 0.017, CHCl_3_), already described [8].

**Scheme 2:**
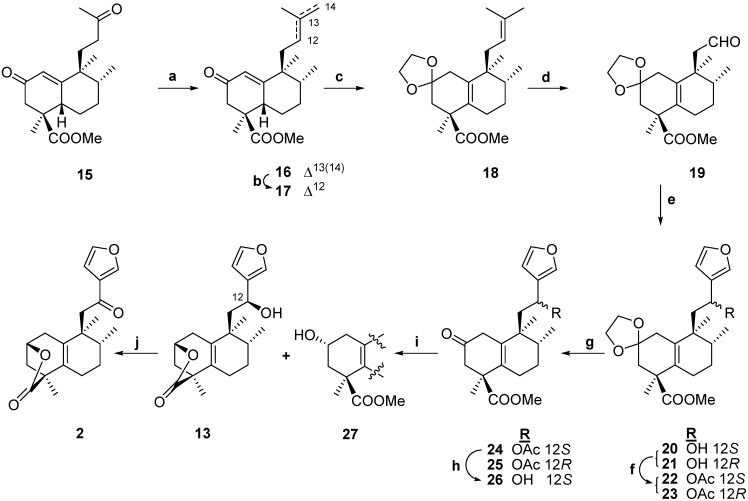
a) MePPh_3_Br, NaHMDS, THF, -78º C, 30 min (94%); b) *p*-TsOH, C_6_H_6_, 60º C, 2h (96%); c) (CH_2_OH)_2_, *p*-TsOH, C_6_H_6_, Dean Stark, 16 h, (97%), d) 1.- OsO_4_, NMO, *^t^*BuOH, THF, H_2_O, rt, overnight; 2.- Pb(AcO)_4_, C_6_H_6_, rt, 39 min (96%, two steps); e) 3-bromofuran, *n*-BuLi, THF, 30 min (**20** : 54%, **21** : 39%); f) Ac_2_O, pyridine 12 h (98%); g) HCl·2M, EtOH, rt, 3 h (96%); h) Na_2_CO_3_, MeOH, 2 h, (96%); i) NaBH_4_, EtOH, rt, 3h (**13**: 38%, **27**: 43%); j) TPAP, NMO, DCM, rt, 50 min (92%).

Route B (Scheme 2) involves first a new procedure for the synthesis of the key intermediate, the tetranorderivative **19 **already used by our group in the synthesis of chetaphanin I and II, and secondly the transformation of this intermediate into the natural compound **2**. Our new route for the synthesis of intermediate **19** gives a better global yield than one based on a Baeyer-Villiger reaction as a key step [4, 6], and can be done in a multigram scale.

Starting from **15**, previously obtained from *ent*-halimic acid, **1 **[4], by chemoselective Wittig reaction [15,16,17], we obtained **16**. Treatment of **16** under acidic conditions allows the isomerization of the side chain terminal double bond into the more stable trisusbstituted position in quantitative yield, to give compound **17**. The protection of the carbonyl group gives the dioxolane **18**, with concomitant isomerization of the double bond in the bicyclic system to the tetrasusbsituted position. Chemoselective oxidation of the side chain of **18** was achieved by treatment with OsO_4 _[18,19,20,21], followed by cleavage of the resulting diol with Pb(AcO)_4_ to give aldehyde **19** in excellent overall yield (84%) from *ent*-halimic acid **1**. In this manner we have opened a new and versatile route to intermediate **19**, a key compound for the synthesis of many natural products. Once this compound was available in large quantities in a reliable fashion it was decided to synthesize compound **2**.

Reaction of **19** with 3-furyl lithium gives **20 **and **21**, that were separated and charactherised as their acetyl derivatives **22** and **23**. The careful hydrolysis in acid medium of **22 **and **23** led to ketones **24 **and **25**. Alkaline hydrolysis of **24 **gives the hydroxyderivative **26**. Reduction of **26 **with NaBH_4_ produced **27** and lactone **13.** Oxidation of **13** gives the desired *ent*-halimanolide **2** 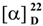 -101.4 (c 0.2, CHCl_3_). The overall yield for the synthesis of *ent*-halimanolide **2** from *ent*-halimic acid **1** by route B was 26%. Compound **2** has been tested against human tumor cell lines: HL-60, IC_50_ >10^-5^M, HeLa, IC_50_, 1.6±0.1 10^-5^M, A549, IC_50_ >10^-5^M, HT-29, IC_50_ >10^-5^M. As can be seen, compound **2**, is only moderately active against HeLa (human cervix cancer).

## Conclusions

The synthesis of the natural *ent*-halimanolide **2** has been achieved starting from *ent*-halimic acid **1**, confirming in this way its structure and establishing its absolute configuration. A new and versatile route to the key intermediate 19 [84% from 1] for the synthesis of natural *ent*-halimanolides is described. Other biological tests for **2** and several intermediates are in progress and will be reported in due course.

## Experimental

### General

Unless otherwise stated, all chemicals were purchased were of the highest purity commercially available and were used without further purification. IR spectra were recorded on a BOMEM 100 FT-IR or an AVATAR 370 FT-IR Thermo Nicolet spectrophotometers. ^1^H- and ^13^C-NMR spectra were recorded for CDCl_3_ solutions and referenced to the residual peak of CHCl_3_ at δ 7.26 ppm and δ 77.0 ppm, for ^1^H and ^13^C, respectively, using Varian 200 VX and Bruker DRX 400 instruments. Chemical shifts are reported in δ ppm and coupling constants (*J*) are given in Hz. MS were performed at a VG-TS 250 spectrometer at 70 eV ionising voltage. Mass spectra are presented as *m/z* (% rel. int.). HRMS were recorded on a VG Platform (Fisons) spectrometer using chemical ionization (ammonia as gas) or Fast Atom Bombardment (FAB) technique. For some of the samples, QSTAR XL spectrometer was employed for electrospray ionization (ESI). Optical rotations were determined on a Perkin-Elmer 241 polarimeter in 1 dm cells. Diethyl ether and THF were distilled from sodium, and dichloromethane was distilled from calcium hydride under Ar atmosphere.

### Methyl 12-acetoxy-2-ethylenedioxy-13,14,15,16-tetranor-ent-halim-5(10)-en-18-oate (**7**)

Acetate **6** (1.02 g, 3.28 mmol) dissolved in benzene (33 mL), was refluxed in the presence of *p*‑toluenesulfonic acid (17 mg, 0.10 mmol) and ethylene glycol (2.0 mL, excess) at 138 ºC for 16 h, then the solution was diluted with AcOEt, washed with 6% aqueous NaHCO_3_ and water and dried over Na_2_SO_4_. The solvent was evaporated to yield **7** (546 mg, 97%) as a colourless oil; 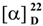-19.1 (c 1.3, CHCl_3_); IR (film) ν (cm^-1^) 1738, 1458, 1373, 1238, 1080, 1032; ^1^H-NMR (200 MHz): 4.20-3.80 (6H, m, -OC_2_H_4_O-, H-12), 3.63 (3H, s, -COOMe), 2.41 (1H, d, *J* = 13.2 Hz, H_A_-3), 2.24 (2H, s, H-1), 2.01 (3H, s, MeCOO-), 1.80-1.50 (4H, m), 1.72 (1H, d, *J* = 13.2 Hz, H_B_-3), 1.50-1.20 (3H, m), 1.33 (3H, s, Me-19), 0.88 (3H, s, Me-20), 0.87 (3H, d, *J* = 6.7 Hz, Me-17); ^13^C-NMR (50 MHz): 177.2 (C-18), 170.9 (MeCOO-), 132.7 (C-10), 130.9 (C-5), 107.5 (C-2), 64.3/64.1 (-OC_2_H_4_O-), 61.6 (C-12), 51.9 (-COOMe), 48.9 (C-4), 42.0 (C-3), 39.8 (C-9), 35.8 (C-1), 35.6 (C-11), 34.1 (C-8), 26.5 (C-7), 24.9 (C-6), 23.7 (C-19), 20.9 (C-20), 20.8 (MeCOO-), 15.9 (C-17); HRMS (EI) m/z calcd. for C_21_H_32_O_6_ (M)^+^: 380.2199; found 380.2191.

### Methyl 12-acetoxy-2-oxo-13,14,15,16-tetranor-ent-halim-5(10)-en-18-oate (**8**)

To a solution of acetate **7 **(48 mg, 0.13 mmol) in EtOH (2.0 mL), aq. HCl (2M, 1.7 mL) was added. The reaction mixture was stirred for 3 h at room temperature, then it was diluted with Et_2_O, extracted with Et_2_O, washed with water and dried over Na_2_SO_4_. Evaporation of the organic layer yielded **8** (41 mg, 92%) as a colourless oil; 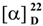-9.8 (c 1.0, CHCl_3_); IR (film) ν (cm^-1^) 2963, 1736, 1680, 1459, 1238, 1033; ^1^H-NMR (200 MHz): 4.09-3.95 (1H, m, H-12), 3.83-3.74 (1H, m, H-12), 3.68 (3H, s, -COOMe), 2.92 (1H, d, *J* = 12.2 Hz, H_A_-1), 2.90 (1H, d, *J* = 15.0 Hz, H_A_-3), 2.25(1H, d, *J* = 15.0 Hz, H_B_-3), 2.15-1.98 (3H, m), 2.01 (3H, s, MeCOO-), 1.92-1.49 (5H, m), 1.27 (3H, s, Me-19), 0.90 (3H, d, *J* = 6.6 Hz, Me-17), 0.86 (3H, s, Me-20);^13^C-NMR (50 MHz): 208.2 (C-2), 174.3 (C-18), 170.7 (MeCOO-), 133.3 (C-10), 133.0 (C-5), 60.7 (C-12), 52.0 (-COOMe), 48.9 (C-1), 48.4 (C-4), 39.4 (C-9), 38.8 (C-3), 34.2 (C-11), 33.3 (C-8), 26.0 (C-7), 25.1 (C-6), 21.8 (C-19), 20.5 (C-20), 20.2 (MeCOO-), 15.4 (C-17); HRMS (EI) m/z calcd. for C_19_H_28_O_5_(M^+^): 336.1937; found 336.1928.

### 12-Acetoxy-13,14,15,16-tetranor-ent-halim-5(10)-en-18,2β-olide (**10**) and methyl 12-acetoxy-2R-hydroxy-13,14,15,16-tetranor-ent-halim-5(10)-en-18-oate (**9**)

To an ice cooled solution of **8** (0.15 g, 0.43 mmol) in EtOH (4.3 mL), NaBH_4_ (16.2 mg, 0.43 mmol) was added. After being stirred at room temperature for 3h, the reaction mixture was recooled to 0ºC and quenched with a few drops of 2 M aqueous HCl solution, diluted with EtOAc and water and extracted with EtOAc. The organic layer was washed with water. Evaporation of the dried extract gave a residue which was chromatographed on silica gel (hex/EtOAc 9:1) to afford **10** (73 mg, 49%) and **9 **(70 mg, 47%). *Compound* **10**: a colourless oil; 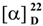-103.1 (c 0.4, CHCl_3_); IR (film) ν (cm^-1^) 2959, 1769, 1732, 1456, 1238, 1081,1031; ^1^H-NMR (400 MHz): 4.81 (1H, m, H-2), 4.03 (1H, m H-12), 3.84 (1H, m, H-12), 2.61-2.24 (4H, m), 2.15-2.01 (3H, m), 2.02 (3H, s, OCOMe), 1.95- 1.69 (4H, m), 1.30 (3H, s, Me-19), 0.91 (3H, s, Me-20), 0.88 (3H, d, *J* = 7.0 Hz, Me-17); ^13^C-NMR (100 MHz): 171.10 (MeCOO-), 169.41 (C-18), 133.3 (C-10), 133.0 (C-5), 74.2 (C-2), 60.9 (C-12), 43.4 (C-4), 41.1 (C-1), 39.0 (C-9), 36.1 (C-3), 33.0 (C-8), 31.2 (C-11), 25.9 (C-7), 23.8 (C-6), 21.6 (C-19), 20.8 (MeCOO-), 16.8 (C-20), 20.2 (MeCOO-), 15.5 (C-17); HRMS (EI) m/z calcd. for C_18_H_26_O_4_Na 329.1723; found 329.1716; *Compound* **9**: a colourless oil; 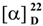-88.0 (c 0.7, CHCl_3_); IR (film) ν (cm^-1^) 3440, 2938, 1732, 1460, 1368, 1273, 1162, 1045; ^1^H-NMR (200 MHz): 4.01 (3H, m, H-2, H-12), 3.64 (3H, s, -COOMe), 2.48-2.10 (4H, m), 2.01 (3H, s, MeCOO-), 1.85-1.40 (6H, m), 1.30 (3H, s, Me-19), 0.90 (3H, s, Me-20), 0.87 (3H, d, *J* = 7.0 Hz, Me-17); ^13^C-NMR (50 MHz): 176.8 (C-18), 170.7 (MeCOO-), 132.7 (C-10), 130.1 (C-5), 65.0 (C-2), 61.3 (C-12), 51.7 (-COOMe), 48.2 (C-4), 43.9 (C-1), 39.3 (C-9), 36.2 (C-3), 33.6 (C-8), 34.2 (C-11), 25.8 (C-7), 24.6 (C-19), 23.4 (C-6), 20.7 (MeCOO-), 20.7 (C-20), 20.7 (MeCOO-), 15.4 (C-17); HRMS (EI) m/z calcd. for C_19_H_30_O_5_Na 361.1985; found 361.1976.

### 12-Hydroxy-13,14,15,16-tetranor-ent-halim-5(10)-en-18,2β-olide (**11**)

To **10** (73.0 mg, 0.24 mmol) a 3% solution of K_2_CO_3_ in methanol (5 ml) was added. After 2 h the solvent was evaporated and diluted with Et_2_O. The organic layer was successively washed with a 2N aqueous solution of HCl and water, dried over Na_2_SO_4_ and evaporated to yield the expected compound **11** (61 mg, 97%) as a colourless oil; 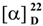-105.5 (c 0.4, CHCl_3_); IR (film) ν (cm^-1^) 3419, 2938, 1770, 1457, 1381, 1189, 1162, 948; ^1^H-NMR (200 MHz): 4.82 (1H, m, H-2), 3.67-3.48 (1H, m, H_A_-12), 3.42-3.28 (1H, m, H_B_-12), 2.45-2.05 (5H, m), 1.96-1.43 (5H, m), 1.28 (3H, s, Me-19), 0.88 (3H, s, Me-20), 0.86 (3H, d, *J* = 6.1 Hz, Me-17); ^13^C-NMR (50 MHz): 179.2 (C-18), 134.0 (C-10), 133.3 (C-5), 74.6 (C-2), 59.2 (C-12), 43.7 (C-4), 41.3 (C-1), 40.3 (C-3), 39.3 (C-9), 33.7 (C-8), 31.4 (C-11), 26.4 (C-6), 24.5 (C-7), 21.8 (C-20), 17.1 (C-19), 16.0 (C-17); HRMS (EI) m/z calcd. for C_16_H_24_O_3_Na 287.1618; found 287.1612.

### 12-Oxo-13,14,15,16-tetranor-ent-halim-5(10)-en-18,2β-olide (**12**)

To a mixture of **11** (32 mg, 0.12 mmol) *N*-methylmorpholine-*N*-oxide (NMO) (49 mg, 0.36 mmol) and molecular sieves (60 mg, 500 mg/mmol) in anhydrous CH_2_Cl_2 _(1.2 mL) under an Ar atmosphere and at room temperature, TPAP (2 mg, 5x10^-3^ mmol) was added. The reaction mixture was stirred for 30 min. and then filtered on silica gel and Celite (DCM and EtOAc). Evaporation of the solvent yielded **12** (30 mg, 96%) as a colourless oil, IR (film) ν (cm^-1^) 2937, 1770, 1719, 1460, 1189,1078,948; ^1^H-NMR (200 MHz): 9.56 (1H, m, H-12), 4.80 (1H, m, H-2), 2.48-2.41 (3H, m), 2.19-2.14 (2H, m), 1.96 (1H, d, *J* = 13.0 Hz, H-11_B_), 1.77- 1.20 (5H, m), 1.30 (3H, s, Me-19), 1.02 (3H, s, Me-20), 0.90 (3H, d, *J* = 6.6 Hz, Me-17); HRMS (EI) m/z calcd. for C_16_H_22_O_3_Na 285.1461; found 281.1476.

### 15,16-Epoxy-12S-hidroxy-ent-halima-5(10),13(16),14-trien-18,2β-olide (**13**) and 15,16-epoxy-12R-hydroxy-ent-halima-5(10),13(16),14-trien-18,2β-olide (**14**)

A solution of 3-bromofuran in THF 1M (0.14 mL, 0.14 mmol), was treated dropwise with *n*-BuLi (1.6 M in hexane, 0.09 mL, 0.14 mmol) at –78^o^C under Ar atmosphere. After the reaction mixture was stirred for 30 min. at this temperature, a solution of **12** (30 mg, 0.11 mmol) in dry THF (1.1 mL) was added and stirred for an additional 30 minutes at the same temperature. The reaction mixture was treated with 10% aqueous NH_4_Cl solution, warmed to room temperature and extracted with EtOAc. The organic layer was washed with 6% NaHCO_3_, brine and dried over Na_2_SO_4_. The solvent was evaporated to afford a residue which was purified by chromatography (Hex/AcOEt 9/1) to yield **13 **(20 mg, 54%) and **14 **(13 mg, 36%). *Compound* **13**: colourless oil; [α]-31.1 (c 0.4, CHCl_3_). IR (film) ν (cm^-1^) 3400, 2927, 1769, 1460, 1070, 1024. ^1^H-NMR (200 MHz): 7.39 (1H, s, H-15), 7.36 (1H, s, H-16), 6.40 (1H, d, *J* = 2.20 Hz, H-14), 4.76 (1H, dd, *J* = 5.6, 2.7 Hz, H-2), 4.43 (1H, d, *J* = 7.9 Hz, H-12), 2.38-1.93 (7H, m), 1.91-1.42 (4H, m), 1.30 (3H, s, Me-19), 0.92 (3H, d, *J* = 6.6 Hz, Me-17), 0.91(3H, s, Me-20). ^13^C-NMR (50 MHz): 178.6 (C-18),143.5 (C-16), 138.5 (C-15), 134.0 (C-10), 133.6 (C-5), 130.9 (C-13), 108.0 (C-14), 74.5 (C-2), 64.0 (C-12), 45.3 (C-11), 43.3 (C-4), 41.0 (C-3), 40.4 (C-9), 33.0 (C-8), 31.1 (C-1), 26.0 (C-7), 24.1 (C-6), 21.1 (C-20), 16.9 (C-19), 15.8 (C-17); HRMS (EI) m/z calcd. for C_20_H_26_O_4_Na: 353.1723; found 353.1718. *Compound* **14**: colourless oil; 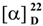-63.1 (c 0.3, CHCl_3_); IR (film) ν (cm^-1^) 3407, 2927, 1769, 1460, 1075, 1024; ^1^H-NMR (200 MHz): 7.40 (1H, d, *J* = 1.6 Hz, H-15), 7.36 (1H, s, H-16), 6.41 (1H, dd, *J* = 2.13, 0.61 Hz, H-14), 4.76 (1H, ddd, *J* = 5.6, 2.9, 2.7 Hz, H-2), 4.44 (1H, dd, *J* = 7.7, 1.7 Hz, H-12), 2.34-1.93 (7H, m), 1.90-1.40 (4H, m), 1.32 (3H, s, Me-19), 0.94 (3H, d, *J* = 6.8 Hz, Me-17), 0.93 (3H, s, Me-20); ^13^C-NMR (50 MHz): 178.7 (C-18),143.5 (C-16), 138.7 (C-15), 134.0 (C-10), 133.7 (C-5), 130.7 (C-13), 108.2 (C-14), 74.3 (C-2), 64.2 (C-12), 45.6 (C-11), 43.5 (C-4), 41.2 (C-3), 40.5 (C-9), 33.1 (C-8), 31.4 (C-1), 26.2 (C-7), 24.3 (C-6), 21.4 (C-20), 16.9 (C-19), 15.8 (C-17); HRMS (EI) m/z calcd. for C_20_H_26_O_4_Na: 353.1723; found 353.1712.

### 15,16-Epoxy-12-oxo-ent-halima-5(10),13(16),14-trien-18,2β-olide (**2**)

To a mixture of **13/14** (3 mg, 0.01 mmol) *N*-methylmorpholine-*N*-oxide (NMO, 4 mg, 0.03 mmol) and molecular sieves (10 mg) in anhydrous CH_2_Cl_2 _(0.3 mL) under an Ar atmosphere and at room temperature, TPAP (1.0 mg, 3x10^-3^ mmol) was added. The reaction mixture was stirred for 30 min. and then filtered on silica gel and Celite (DCM and EtOAc). Evaporation of the solvent yielded **2** (3.0 mg, 92%) as a colourless oil; 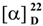-101.4 (c 0.2, CHCl_3_); IR (film) ν (cm^-1^) 2928, 1733, 1240, 1077, 1023; ^1^H-NMR (200 MHz): 7.94 (1H, dd, *J* = 1.6, 0.8 Hz, H-16), 7.41 (1H, dd, *J* = 12.0, 1.6 Hz, H-15), 6.73 (1H, dd, *J* = 1.6, 0.8 Hz, H-14), 4.76 (1H, ddd, *J* = 5.6, 2.8, 2.8 Hz, H-2), 2.85 (1H, d, *J* = 15.6 Hz, H_A_-11), 2.74 (1H, d, *J* = 15.6 Hz, H_B_-11), 2.39-2.35 (2H, m, H-1), 2.19-2.10 (2H, m, H-6), 2.13 (1H, dd, *J* = 10.8, 6.8 Hz, H_A_-3), 2.08-2.04 (1H, m, H-8), 1.94 (1H, d, *J* = 10.8 Hz, H_B_-3), 1.81-1.42 (2H, m, H-7), 1.32 (3H, s, Me-19), 1.09 (3H, s, Me-20), 0.86 (3H, d, *J* = 7.0 Hz, Me-17); ^13^C-NMR (50 MHz): 193.5 (C-12), 178.2 (C-18), 146.9 (C-16), 144.1 (C-15), 132.4 (C-10), 132.1 (C-5), 129.3 (C-13), 108.7 (C-14), 73.9 (C-2), 47.7 (C-11), 43.5 (C-4), 41.1 (C-3), 40.3 (C-9), 33.2 (C-8), 31.6 (C-1), 25.2 (C-7), 22.1 (C-6), 21.8 (C-20), 16.4 (C-19), 15.1 (C-17); HRMS (EI) m/z calcd. for C_20_H_24_O_4_Na: 351.1567; found 351.1567.

### Methyl 2-oxo-15-nor-ent-halima-1(10),13(14)-dien-18-oate (**16**)

To a suspension of MeOCH_2_PPh_3_Cl (1.24 g, 3.15 mmol) in THF (10 mL) at –20^o^C under an Ar atmosphere, 1.0 M NaHMDS in THF (3.15 mL, 3.15 mmol) was added dropwise and the solution was stirred for 30 min. A solution of the aldehyde **15** (1.0 g, 3.12 mmol) in THF (15 mL) was added dropwise at –78ºC. The mixture was stirred for 1 h at room temperature. Then, it was quenched with aqueous NH_4_Cl and extracted with AcOEt. The organic layer was washed with brine and dried over Na_2_SO_4_. The solvent was evaporated to afford a residue which was purified by chromatography (Hex/ AcOEt 97/3) to yield **16** (933 mg, 94%) as a colourless oil; 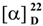
+179.9 (c 1.4, CHCl_3_); IR (film) ν (cm^-1^) 2964, 1732, 1676, 1455, 1267, 1160, 1113, 883;^ 1^H-NMR (200 MHz): 5.81 (1H, s, H-1), 4.68 (2H, s, H-14), 3.60 (3H, s, -COOMe), 3.00 (1H, dd, *J* = 12.1, 4.7 Hz, H-5), 2.66 (1H, d, *J* = 16.2 Hz, H-3_A_), 2.24 (1H, d, *J* = 16.2 Hz, H-3_B_), 2.18-1.97 (3H, m), 1.83-1.36 (6H, m), 1.70 (3H, s, Me-16), 1.20 (3H, s, Me-19), 0.98 (3H, s, Me-20), 0.78 (3H, d, *J* = 7.4 Hz, Me-17); ^13^C-NMR (50 MHz): 196.3 (C-2), 175.9 (C-18) 168.7 (C-10), 145.2 (C-13), 124.2 (C-1), 108.9 (C-14), 51.6 (-COOMe), 45.5 (C-4), 44.4 (C-9), 42.2 (C-3), 40.2 (C-5), 39.9 (C-8), 36.4 (C-12), 31.1 (C-11), 27.5 (C-7), 22.8 (C-6), 21.9 (C-19), 21.2 (C-16), 20.0 (C-20), 14.8 (C-17); HRMS (EI) m/z calcd. for C_20_H_30_O_3_Na: 341.2087; found 341.2073.

### Methyl 2-oxo-15-nor-ent-halima-1(10),12(13)-dien-18-oate (**17**)

To a solution of **16** (1.06 g, 3.35 mmol) in benzene (33 mL), *p*-TsOH (0.16 g, 0.91 mmol) was added. The reaction mixture was stirred at 60ºC for 2 h, then it was cooled and diluted with Et_2_O. The organic layer was washed with 6% aqueous NaHCO_3_ and brine and dried over Na_2_SO_4_. The solvent was evaporated to yield **17** (1.01 g, 96%) as a colourless oil; 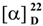
+45.4 (c 1.0, CHCl_3_); IR (film) ν (cm^-1^) 2929, 1732, 1676, 1457, 1269, 1116; ^1^H-NMR (200 MHz): 5.74 (1H, s, H-1), 4.71 (1H, m, H-12), 3.57 (3H, s, -COOMe), 3.00 (1H, dd, *J* = 12.4, 4.5 Hz, H-5), 2.63 (1H, d, *J* = 16.0 Hz, H-3_A_), 2.19 (1H, d, *J* = 15.6 H-3_B_), 2.10-1.90 (3H, m), 1.85- 1.22 (5H, m), 1.53 (3H, s, Me-16), 1.47 (3H, s, Me- 14), 1.16 (3H, s, Me-19), 0.86 (3H, s, Me-20), 0.72 (3H, d, *J* = 7.0 Hz, Me-17); ^13^C-NMR (50 MHz): 197.4 (C-2), 176.8 (C-18) 169.8 (C-10), 133.9 (C-13), 124.9 (C-1), 120.0 (C-12), 52.5 (-COOMe), 46.5 (C-4), 46.1 (C-9), 43.6 (C-3), 41.2 (C-5), 40.3 (C-8), 37.7 (C-11), 28.6 (C-7), 26.2 (C-20), 23.7 (C-6), 21.7 (C-19), 21.7 (C-16), 18.2 (C-14), 15.8 (C-17); HRMS (EI) m/z calcd. for C_20_H_30_O_3_Na: 341.2087; found 341.2097.

### Methyl 2-ethylenedioxy-15-nor-ent-halima-5(10),12(13)-dien-18-oate (**18**)

Compound **17** (1 g, 3.15 mmol) dissolved in benzene (32 mL), was refluxed in the presence of *p*‑toluenesulfonic acid (19 mg, 0.11 mmol) and ethylene glycol (1.9 ml, excess) at 138ºC for 16 h. The solution was then diluted with AcOEt and washed with 6% aqueous NaHCO_3_ and water and dried over Na_2_SO_4_. The solvent was evaporated to yield **18** (1.11 g, 97%) as a colourless oil; 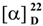-20.3 (c 1.4, CHCl_3_); IR (film) ν (cm^-1^) 2964, 1733, 1237, 1118, 1078; ^1^H-NMR (200 MHz): 4.98 (1H, m, H-12), 3.96 (4H, m, -OC_2_H_4_O-), 3.64 (3H, s, -COOMe), 2.31 (2H, s, H-3), 2.20-1.95 (4H, m), 1.92-1.30(5H, m), 1.66 (3H, s, Me-16), 1.57 (3H, s, Me-14), 1.33 (3H, s, Me-19), 0.84 (3H, s, Me-20), 0.80 (3H, d, *J* = 7.0 Hz, Me-17); ^13^C-NMR (50 MHz): 176.1 (C-18), 132.5 (C-13), 131.0 (C-5), 129.2 (C-10), 120.6 (C-12), 106.7 (C-2), 63.2 (-OC_2_H_4_O-), 63.0 (-OC_2_H_4_O-), 50.7 (-COOMe), 48.0 (C-4), 40.8 (C-3), 39.8 (C-9), 34.8 (C-1), 34.7 (C-11), 32.8 (C-8), 25.6 (C-7), 25.1 (C-20), 23.9 (C-6), 22.6 (C-16), 19.4 (C-19), 17.0 (C-14), 15.2 (C-17); HRMS (EI) m/z calcd. for C_22_H_34_O_4_Na: 385.2349; found 385.2362.

### Methyl 2-ethylenedioxy-12-oxo-13,14,15,16-tetranor-ent-halim-5(10)-en-18-oate (**19**)

To a solution of **18** (1.05 g, 2.90 mmol) in *t*-BuOH/THF/H_2_O (7:2:1, 30.5 ml) was added *N*-methyl-morpholine *N*-oxide (NMO, 1.18 g, 8.70 mmol) and a solution of OsO_4_ 2.5% (0.3 ml, 0.01mmol) in *t*‑BuOH. The reaction mixture was stirred at room temperature for 20 h and a saturated aqueous solution of Na_2_SO_3_ (30 mL) was added. The mixture was extracted with AcOEt, and the organic layer was washed with 10% aqueous Na_2_S_2_O_3_, 2N aqueous HCl, water and brine and dried over Na_2_SO_4_. The solvent was evaporated to yield the expected mixture of hydroxy derivatives. To a solution of the hydroxy derivatives (1.15g, 3.06 mmol) in benzene (16 ml) was added LTA (3.0 g, 6.68 mmol). The reaction mixture was stirred at room temperature for 30 min and then filtered off through Celite. The solution was diluted with EtOAc and washed with 6% aqueous NaHCO_3_, water and brine and then dried and evaporated to yield **19 **(980 mg, 96%) as a colourless oil; 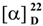
+2.1 (c 0.9, CHCl_3_); IR (film) ν (cm^-1^) 1734, 1717, 1456, 1375, 1238, 1152, 1078, 1032; ^1^H-NMR (200 MHz): 9.63 (1H, s, H-12), 4.00-3.80 (4H, m, -OC_2_H_4_O-), 3.60 (3H, s, -COOMe), 2.60-2.20 (5H, m, H-11, H-1, H_A_-3), 2.10-1.90 (1H, m), 1.62 (1H, d, *J* = 13.2 Hz, H_B_-3), 1.80-1.60 (2H, m), 1.40-1.20 (2H, m), 1.31 (3H, s, Me-19), 0.96 (3H, s, Me-20), 0.88 (3H, d, *J* = 6.7 Hz, Me-17); ^13^C-NMR (50 MHz): 204.3 (C-12), 177.0 (C-18), 131.7 (C-10), 131.3 (C-5), 107.2 (C-2), 64.3/64.1 (-OC_2_H_4_O-), 51.9 (-COOMe), 51.2 (C-11), 48.8 (C-4), 42.2 (C-3), 40.1 (C-9), 36.4 (C-1), 36.4 (C-8), 26.2 (C-7), 24.5 (C-6), 23.9 (C-19), 21.1 (C-20), 15.7 (C-17); HRMS (EI) m/z calcd. for C_19_H_28_O_5_ (M)^+^: 336.1937; found 336.1916.

### Methyl 15,16-epoxy-2-ethylenedioxy-12S-hydroxy-ent-halima-5(10),13(16),14-trien-18-oate (**20**) and methyl 15,16-epoxy-2- ethylenedioxy -12R-hydroxy-ent-halima-5(10),13(16),14-trien-18-oate (**21**)

A solution of 3-bromofuran in THF 0.89M (1.45 mL, 1.28 mmol), was treated dropwise with *n*‑BuLi (1.6 M in hexane, 0.85 ml, 1.35 mmol) at –78^o^C under an Ar atmosphere. After the reaction mixture was stirred for 30 minutes at this temperature, a solution of **19** (430 mg, 1.28 mmol) in dry THF (1.1 mL) was added and stirred for an additional 30 min. at the same temperature. The reaction mixture was then treated with 10% aqueous NH_4_Cl solution, warmed to room temperature and extracted with EtOAc. The organic layer was washed with 6% NaHCO_3_, brine and dried over Na_2_SO_4_. The solvent was evaporated to afford a residue which was purified by chromatography (hex/AcOEt 95/5) to yield **20 **(279 mg, 54%) and **21 **(202 mg, 39%). *Compound* **20**: a colourless oil; 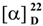
+4.6 (c 0.8, CHCl_3_); IR (film) ν (cm^-1^) 3500, 1724, 1458, 1375, 1262, 1157, 1078, 1030, 665; ^1^H-NMR (200 MHz): 7.34 (2H, s, H-15, H-16), 6.39 (1H, s, H-14), 4.85 (1H, dd, *J* = 9.0, 2.2 Hz, H-12), 4.00-3.80 (4H, m, -OC_2_H_4_O-), 3.67 (3H, s, -COOMe), 2.38 (1H, d, *J* = 12.5 Hz, H_A_-3), 2.35 (1H, d, *J* = 10.5 Hz, H_A_-1), 2.15 (1H, d, *J* = 10.5 Hz, H_B_-1), 2.00 (1H, dd, *J* = 15.0, 9.4 Hz, H_A_-11), 1.72 (1H, dd, *J* = 15.0, 2.2 Hz, H_B_-11), 1.71-1.68 (2H, m, H-6), 1.70 (1H, d, *J* = 12.5 Hz, H_B_-3), 1.63-1.56 (1H, m, H-8), 1.37-1.27 (2H, m, H-7), 1.35 (3H, s, Me-19), 0.93 (3H, s, Me-20), 0.92 (3H, d, *J* = 6.8 Hz, Me-17); ^13^C-NMR (50 MHz): 177.6 (C-18), 143.0 (C-16), 138.2 (C-15), 133.2 (C-10), 131.7 (C-5), 130.8 (C-13), 108.6 (C-14), 107.3 (C-2), 64.4 (C-12), 64.4/64.2 (-OC_2_H_4_O-), 52.0 (-COOMe), 48.6 (C-4), 46.5 (C-11), 42.7 (C-3), 40.6 (C-9), 36.7 (C-1), 35.3 (C-8), 26.8 (C-7), 25.2 (C-6), 24.5 (C-19), 21.3 (C-20), 16.1 (C-17); HRMS (EI) m/z calcd for C_23_H_32_O_6_ (M)^+^: 404.2199; found 404.2191. *Compound* **21**: a colourless oil; 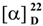-17.2 (c 0.5, CHCl_3_); IR (film) ν (cm^-1^) 3500, 1730, 1464, 1377, 1159, 1089, 1030, 665; ^1^H-NMR (200 MHz): 7.38 (2H, s, H-15, H-16), 6.38 (1H, s, H-14), 4.91 (1H, dd, *J* = 9.4, 2.3 Hz, H-12), 4.00-3.80 (4H, m, -OC_2_H_4_O-), 3.65 (3H, s, -COOMe), 2.60-2.00 (5H, m), 1.90-1.60 (3H, m), 1.40-1.20 (3H, m), 1.36 (3H, s, Me-19), 0.91 (3H, s, Me-20), 0.89 (3H, d, *J* = 6.9 Hz, Me-17); ^13^C-NMR (50 MHz): 177.6 (C-18), 143.0 (C-16), 138.4 (C-15), 133.4 (C-10), 132.0 (C-5), 130.0 (C-13), 108.7 (C-14), 107.4 (C-2), 64.6/64.3 (-OC_2_H_4_O-), 64.2 (C-12), 52.1 (-COOMe), 48.7 (C-4), 47.7 (C-11), 42.9 (C-3), 40.6 (C-9), 36.1 (C-1), 35.8 (C-8), 26.9 (C-7), 25.1 (C-6), 24.5 (C-19), 21.8 (C-20), 16.4 (C-17), HRMS (EI) m/z calcd. for C_23_H_32_O_6_ (M)^+^: 404.2199; found 404.2193.

### Methyl 12S-acetoxy-15,16-epoxy-2-ethylenedioxy-ent-halima-5(10),13(16),14-trien-18-oate (**22**)

To a solution of **20** (118 mg, 0.29 mmol) in dry pyridine (1.0 mL), Ac_2_O (1.0 mL) was added and the mixture was stirred at room temperature overnight, then the reaction mixture was poured into ice-water and extracted with EtOAc. The organic layer was washed successively with 2M aqueous HCl, 6% aqueous NaHCO_3 _and brine. It was dried over Na_2_SO_4 _and the solvent was evaporated to afford **22 **(128 mg, 99%) as a colourless oil; 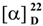-13.3 (c 0.7, CHCl_3_); IR (film) ν (cm^-1^) 1733, 1458, 1374, 1240, 1102, 1077, 1023, 874; ^1^H-NMR (200 MHz): 7.36 (1H, s, H-16), 7.31 (1H, s, H-15), 6.36 (1H, s, H-14), 5.71 (1H, dd, *J* = 7.8, 2.2 Hz, H-12), 4.00-3.80 (4H, m, -OC_2_H_4_O-), 3.69 (3H, s, -COOMe), 2.25 (1H, d, *J* = 13.2 Hz, H_A_-3), 2.19-1.99 (4H, m), 1.96 (3H, s, -OCOMe), 1.95-1.39 (5H, m), 1.66 (1H, d, *J* = 13.2 Hz, H_B_-3), 1.32 (3H, s, Me-19), 0.89 (3H, d, *J* = 6.9 Hz, Me-17), 0.87 (3H, s, Me-20); ^13^C-NMR (50 MHz): 177.6 (C-18), 170.2 (-OCOMe), 143.3 (C-16), 140.2 (C-15), 132.9 (C-10), 131.5 (C-5), 126.7 (C-13), 109.0 (C-14), 107.7 (C-2), 66.4 (C-12), 64.5/64.3 (-OC_2_H_4_O-), 52.2 (-COOMe), 49.0 (C-4), 42.1 (C-3), 41.9 (C-11), 41.1 (C-9), 36.2 (C-1), 33.8 (C-8), 26.4 (C-7), 25.1 (C-6), 24.0 (C-19), 21.6 (-OCOMe), 20.8 (C-20), 16.0 (C-17); HRMS (EI) m/z calcd. for C_25_H_34_O_7_Na: 469.2197; found 469.2197.

### Methyl 12R-acetoxy-15,16-epoxy-2-ethylenedioxy-ent-halima-5(10),13(16),14-trien-18-oate (**23**)

To a solution of **21** (22 mg, 0.05 mmol) in dry pyridine (0.5 mL), Ac_2_O (0.5 mL) was added and the mixture was stirred at room temperature overnight. The reaction mixture was then poured into ice-water and extracted with EtOAc. The organic layer was washed successively with 2M aqueous HCl, 6% aqueous NaHCO_3 _and brine. It was dried over Na_2_SO_4 _and the solvent was evaporated to afford **23 **(128 mg, 99%) as a colourless oil; 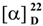-51.8 (c 0.6, CHCl_3_); IR (film) ν (cm^-1^) 1733, 1458, 1374, 1240, 1102, 1077, 1023, 874; ^1^H-NMR (200 MHz): 7.39 (1H, s, H-16), 7.34 (1H, s, H-15), 6.39 (1H, s, H-14), 5.89 (1H, dd, *J* = 8.8, 4.4 Hz, H-12), 3.96-3.80 (4H, m, -OC_2_H_4_O-), 3.66 (3H, s, -COOMe), 2.60-2.00 (5H, m), 2.19-1.99 (4H, m), 2.02 (3H, s, -OCOMe), 1.93-1.62 (3H, m), 1.40-1.20 (3H, m), 1.36 (3H, s, Me-19), 0.87 (3H, s, Me-20), 0.82 (3H, d, *J* = 6.6 Hz, Me-17); ^13^C-NMR (50 MHz): 177.5 (C-18), 170.5 (-OCOMe), 143.3 (C-16), 140.4 (C-15), 133.3 (C-10), 129.7 (C-5), 126.6 (C-13), 109.1 (C-14), 107.9 (C-2), 65.4 (C-12), 64.6/64.2 (-OC_2_H_4_O-), 52.2 (-COOMe), 49.5 (C-4), 41.4 (C-3), 41.2 (C-11), 40.9 (C-9), 36.4 (C-1), 33.4 (C-8), 25.6 (C-7), 25.1 (C-6), 23.6 (C-19), 22.0 (-OCOMe), 21.4 (C-20), 16.1 (C-17); HRMS (EI) m/z calcd. for C_25_H_34_O_7_Na: 469.2197; found 496.2197.

### Methyl 12S-acetoxy-15,16-epoxy-2-oxo-ent-halima-5(10),13(16),14-trien-18-oate (**24**) and methyl 12R-acetoxy-15,16-epoxy-2-oxo-ent-halima-5(10),13(16),14-trien-18-oate (**25**)

To a solution of acetate **22** (118 mg, 0.27 mmol) in EtOH (2.6 mL), aq HCl. (2M, 3.6 mL) was added. The reaction mixture was stirred for 3 h at room temperaure. Then it was diluted and extracted with Et_2_O, washed with water and dried over Na_2_SO_4_. Evaporation of the organic layer yielded **24** (102 mg, 96%) as a colourless oil; 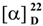-5.0 (c 0.9, CHCl_3_); IR (film) ν (cm^-1^) 2922, 1734, 1717, 1458, 1374, 1234, 1023; ^1^H-NMR (200 MHz): 7.39 (1H, s, H-16), 7.31 (1H, s, H-15), 6.37 (1H, s, H-14), 5.52 (1H, dd, *J* = 7.0, 3.4 Hz, H-12), 3.76 (3H, s, -COOMe), 2.82-2.63 (2H, m), 2.23-2.06 (2H, m), 2.05-1.50 (5H, m), 1.96 (3H, s, -OCOMe), 1.40-1.20 (3H, m), 1.36 (3H, s, Me-19), 0.95 (3H, d, *J* = 6.2 Hz, Me-17), 0.84 (3H, s, Me-20); ^13^C-NMR (50 MHz): 208.9 (C-2), 174.9 (C-18), 170.2 (-OCOMe), 143.6 (C-16), 140.4 (C-15), 134.3 (C-13), 134.0 (C-10), 125.6 (C-5), 108.8 (C-14), 66.0 (C-12), 52.8 (-COOMe), 49.2 (C-1), 48.8 (C-4), 41.3 (C-11), 41.3 (C-9), 39.8 (C-3), 33.9 (C-8), 26.7 (C-7), 26.0 (C-6), 22.2 (-OCOMe), 21.5 (C-19), 20.7 (C-20), 16.0 (C-17); HRMS (EI) m/z calcd. for C_23_H_30_O_6_ Na: 425.1935; found 425.1944.

Similarly, to a solution of acetate **23** (36 mg, 0.08 mmol) in EtOH (0.8 mL), aq HCl. (2M, 1.0 mL) was added. The reaction mixture was stirred for 3 h at room temperature. Then it was diluted with Et_2_O, extracted with Et_2_O, washed with water and dried over Na_2_SO_4_. Evaporation of the organic layer yielded **25** (31 mg, 96%) as a colourless oil; 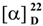-21.1 (c 0.6, CHCl_3_); IR (film) ν (cm^-1^) 2922, 1734, 1718, 1458, 1374, 1234, 1119, 1023; ^1^H-NMR (200 MHz): 7.39 (1H, s, H-16), 7.36 (1H, s, H-15), 6.37 (1H, s, H-14), 5.83 (1H, dd, *J* = 8.4, 4.8 Hz, H-12), 3.72 (3H, s, -COOMe), 3.27 (1H, d, *J* = 21.0 Hz, H_A_-1), 2.99 (1H, d, *J* = 14.2 Hz, H_A_-3), 2.83 (1H, d, *J* = 20.8 Hz, H_B_-1), 2.31 (1H, d, *J* = 14.2 Hz, H_B_-3), 2.20-1.60 (4H, m), 2.01 (3H, s, -OCOMe), 1.50-1.20 (3H, m), 1.24 (3H, s, Me-19), 0.85 (3H, s, Me-20), 0.83 (3H, d, *J* = 6.2 Hz, Me-17); ^13^C-NMR (50 MHz): 209.1 (C-2), 175.1 (C-18), 170.4 (-OCOMe), 143.6 (C-16), 140.4 (C-15), 134.0 (C-13), 132.4 (C-10), 126.2 (C-5), 108.9 (C-14), 65.4 (C-12), 52.6 (-COOMe), 50.5 (C-1), 49.2 (C-4), 40.4(C-9), 40.1 (C-11), 33.3 (C-8), 29.9 (C-3), 26.8 (C-7), 25.7 (C-6), 22.5 (-OCOMe), 22.5 (C-19), 21.8 (C-20), 16.1 (C-17); HRMS (EI) m/z calcd. for C_23_H_30_O_6_Na: 425.1935; found 425.1945.

### Methyl 15,16-epoxy-12S-hydroxy-2-oxo-ent-halima-5(10),13(16),14-trien-18-oate (**26**)

To a solution of **24 **(50 mg, 0.12 mmol) in methanol (1.0 ml) Na_2_CO_3_ (23 mg, 0.21 mmol) was added. The mixture was stirred at room temperature. After 2 h, the solvent was evaporated and diluted with Et_2_O. The organic layer was successively washed with a 2N aqueous solution of HCl and water, dried over Na_2_SO_4_ and evaporated to yield **26** (42 mg, 96%) as a colourless oil; 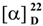-6.1 (c 1.0, CHCl_3_); IR (film) ν (cm^-1^) 3435, 2956, 1724, 1461, 1242, 1120, 1075; ^1^H-NMR (200 MHz): 7.34 (2H, m, H-15, H-16), 6.39 (1H, s, H-14), 4.51 (1H, m, H-12), 3.69 (3H, s, -COOMe), 2.78 (1H, d, *J* = 15.8 Hz, H_A_-3), 2.71 (1H, m, H_A_-1), 2.22 (1H, d, *J* = 15.4 Hz, H_B_-3), 2.19-1.95 (3H, m), 1.93-1.34 (5H, m), 1.25 (3H, s, Me-19), 0.95 (3H, d, *J* = 6.6 Hz, Me-17), 0-85 (3H, s, Me-20); ^13^C-NMR (50 MHz): 207.8 (C-2), 173.9 (C-18), 142.6 (C-16), 137.5 (C-15), 132.8 (C-13), 132.6 (C-10), 129.0 (C-5), 107.2 (C-14), 63.1 (C-12), 51.3 (-COOMe), 48.5 (C-1), 47.7 (C-4), 43.1 (C-11), 39.9 (C-9), 38.5 (C-3), 32.7 (C-8), 25.5 (C-7), 24.6 (C-6), 21.0 (C-19), 19.8 (C-20), 15.0 (C-17); HRMS (EI) m/z calcd. for C_21_H_28_O_5_Na: 383.1829; found 383.1829.

### Methyl 15,16-epoxy-2R,12S-dihydroxy-ent-halima-5(10),13(16),14-trien-18-oate (**27**) and 15,16-epoxy-12S-hydroxy-ent-halima-5(10),13(16),14-trien-18,2β-olide (**13**)

To an ice cooled solution of **26** (22.0 mg, 0.07 mmol) in EtOH (0.7 mL), NaBH_4_ (13 mg, 0.33 mmol) was added. After being stirred at room temperature for 3h, the reaction mixture was recooled to 0ºC and quenched with a few drops of 2 M aqueous HCl solution, diluted with EtOAc and water and extracted with EtOAc. The organic layer was washed with water. Evaporation of the dried extract gave a residue, which was chromatographed on silica gel (hex/EtOAc 9/1) to afford **13** (8 mg, 38%), and **27** (9 mg, 43%). *Compound* **27**: a colourless oil; 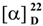-41.1 (c 0.6, CHCl_3_); IR (film) ν (cm^-1^) 3408, 2929, 1727, 1460, 1274, 1161, 1024; ^1^H-NMR (200 MHz): 7.20 (2H, m, H-15, H-14), 6.41 (1H, bs, H-14), 3.97 (2H, m, H-2, H-12), 3.66 (3H, s, -COOMe), 2.42- 1.95 (5H, m), 1.92-1.47 (6H, m), 1.33 (3H, s, Me-19), 0.93 (3H, s, Me-20), 0.85 (3H, d, *J* = 6.6 Hz, Me-17); ^13^C-NMR (50 MHz): 177.9 (C-18), 143.4 (C-16), 138.8(C-15), 133.9 (C-10), 131.4 (C-5), 130.8 (C-13), 108.8 (C-14), 65.5 (C-2), 64.9 (C-12), 52.6 (-COOMe), 49.2 (C-4), 47.3 (C-3), 45.0 (C-1), 40.6 (C-9), 35.4 (C-8), 29.3 (C-7), 26.5 (C-6), 22.1 (C-20), 16.1(C-19), 14.3 (C-17); HRMS (EI) m/z calcd. for C_21_H_30_O_5_ Na: 385.1985; found 385.1969. *Compound* **13**: see above.

### 15,16-Epoxy-12-oxo-ent-halima-5(10),13(16),14-trien-18,2β-olide (**2**)

To a mixture of **13** (3 mg, 0.01 mmol) *N*-methylmorpholine-*N*-oxide (NMO) (4 mg, 0.03 mmol) and molecular sieves (15 mg) in anhydrous CH_2_Cl_2 _(0.3 mL) under an Ar atmosphere and at room temperature, TPAP (3.0 mg, 3x10^-3^ mmol) was added. The reaction mixture was stirred for 30 min. and then filtered on silica gel and Celite (DCM and EtOAc). Evaporation of the solvent yielded **2** (3.0 mg, 92%).
